# Impact of Nurse–Physician Collaboration, Moral Distress, and Professional Autonomy on Job Satisfaction among Nurses Acting as Physician Assistants

**DOI:** 10.3390/ijerph19020661

**Published:** 2022-01-07

**Authors:** Yunmi Kim, Younjae Oh, Eunhee Lee, Shin-Jeong Kim

**Affiliations:** 1 Graduate School of Health Science, Hallym University, Hallymdaehakgil 1, Gangwon-do, Chuncheon 24252, Korea; happy-yunmi@hanmail.net; 2 Kangdong Sacred Heart Hospital, Seongan-ro 150, Gangdong-gu, Seoul 05355, Korea; 3 Research Institute of Nursing Science, College of Nursing, Hallym University, Hallymdaehakgil 1, Gangwon-do, Chuncheon 24252, Korea; ehlee@hallym.ac.kr (E.L.); ksj@hallym.ac.kr (S.-J.K.)

**Keywords:** job satisfaction, moral distress, professional autonomy, collaboration, nurses, physician assistants

## Abstract

Although there is considerable literature on job satisfaction among nurses in various settings, there is little research about contributing factors, including moral distress to job satisfaction among a certain group of nurses, such as nurses acting as physician assistants. The purpose of this study was to verify the impact of nurse–physician collaboration, moral distress, and professional autonomy on job satisfaction among nurses acting as physician assistants. Descriptive and correlational research was conducted on a convenience sample of 130 nurses from five general hospitals in South Korea. In the final regression model, the adjusted R square was significant, explaining 38.2% of the variance of job satisfaction (F = 8.303, *p* < 0.001), where ‘cooperativeness’ (β = 0.469, *p* = 0.001) from nurse–physician collaboration, ‘institutional and contextual factor’ from moral distress (β = −0.292, *p* = 0.014), and professional autonomy (β = 0.247, *p* = 0.015) were included. In hospital environments, a more cooperative inter-professional relationship between nurses and physicians led to less moral distress caused by organisational constraints. A higher level of professional autonomy among nurses acting as physician assistants is required to increase their job satisfaction.

## 1. Introduction

As the healthcare industry has recently expanded its share of the global economy and supported human longevity, the level of public demand for healthcare services has also increased. There is an interest in improving the quality of healthcare. In South Korea, nurses replace their major roles under various titles such as physician assistants (PAs) or medical support nurses to obviate the lack of resident physicians, an imbalance in the supply and demand of resident physicians in major cities, leading to a deterioration in healthcare quality. The history of nurses acting as PAs in South Korea is different from the PAs in the United States; establishing the PA position was an alternative plan for the healthcare of residents in farming and fishing villages, where the supply of medical services has been restricted. In the United Kingdom, with the modification of the PA’s title to a physician associate in 2013, the professional role of a physician associate has been expanded, and a spectrum of advanced healthcare professionals have been required to participate as physician associates, including nurses [1]. For the physician associates, the new healthcare professionals, it is necessary to develop their skills and attitude not only based on the medical model but also on the holistic care approach, such as the nursing care paradigm. For this reason, nurses in the United Kingdom are encouraged to engage as physician associates [2].

The number of nurses acting as PAs has been on the rise since South Korea introduced PAs in 2005; 4136 nurses were acting as PAs in 2019, increasing about fourfold from 1009 in 2010 [3]. However, the roles and scope of responsibility of nurses acting as PAs have not been identified in law or even in institutions where nurses are performing PA work. For instance, nurses acting as PAs can prescribe and perform some of the medical procedures without legal protection; legal and ethical issues regarding their medical practice have been debated in public media recently as a result of a resident physician strike in 2020 [4]. In the media, several nurses acting as PAs were outraged that they had no choice but to engage in PA work by their hospital HR system. They are often anxious about their practice and patient outcomes because of insufficient legal protection and unspecified professional positions. Moreover, nurses’ professional self-concept is at risk due to confusion regarding the central role of their profession between that of nursing and acting PA, resulting in role conflict [5,6]. These conflicts can cause nurses who are functioning as PAs to feel psychological withdrawal and discomfort, exhaustion, increased job stress, and decreased job satisfaction [7,8].

Job satisfaction is defined as the degree of positive emotion expressed by organisational members toward their tasks [9], and job satisfaction is shown to affect the quality of nursing care [10]. Numerous studies have verified various factors and outcomes related to job satisfaction among nurses over the last few decades [11,12]. Professional autonomy, one of the factors affecting job satisfaction [10], is also considered essential to developing nursing professionalism, providing for independent nursing, and improving nursing quality [13,14]. Nurses can be satisfied with their profession by acting independently and possessing creative responsibility for determining, practising, and taking charge of care according to conscience, ethics, law and accountability, with reference to performance standards [10,14]. Nurses’ higher professional autonomy has been shown to increase job satisfaction because it promotes organisational coherence by triggering motivation to provide the best care [15,16]. For nurses acting as PAs in South Korea, unfortunately, the basis for independently making decisions is ambiguous, and legal and ethical responsibilities are not clear, due to an unclear division of duties and the absence of work guidelines [11,13]. Consequently, the job satisfaction of nurses acting as PAs is low compared to general staff nurses [7,8]. It is necessary to identify the relationship between professional autonomy and job satisfaction among nurses acting as PAs.

Meanwhile, a good relationship with physicians is essential because nurses’ professional autonomy is to cooperatively solve problems by forming relationships on an equal footing with other healthcare professionals. Nurse–physician collaboration can be defined as an interaction where nurses and doctors can use their particular expertise and skills to create synergies in nursing care and treatment [17]. The collaborative relationship between nurses and physicians not only increases their job satisfaction but also increases patient satisfaction and is essential to positive treatment outcomes, thus improving the quality of healthcare [18,19]. This collaboration between nurses and physicians is also crucial in ethical decision-making processes that require interdisciplinary understanding. It has become challenging to solve the complex and sensitive ethical problems in the healthcare environment. Thus, it is necessary to communicate and cooperate with health- care team members to appreciate various perspectives and cultures. However, when facing ethical issues, nurses acting as PAs experience difficulties in communication with physicians, a lack of self-esteem, and role conflicts [6,8]. Such experiences can also cause moral distress to nurses. 

Moral distress refers to a painful feeling or extreme psychological discomfort experienced by nurses where they are aware of the right actions but are forced to act contrary to their ethical beliefs due to situational constraints, such as an adverse ethical climate at the hospital, limitations of resources, or a lack of authority in nursing [20,21]. The higher the cooperative relationship with physicians and the higher the professional autonomy of nurses, the less frequent and intense would be the nurses’ experience of moral distress [22,23]. However, in an organisational climate in which the healthcare environment is ranked or in which physicians’ authority is dominant, such as in Asian countries, moral distress can be experienced because nurses are restricted to act in accordance with their ethical principles [24,25]. Furthermore, recent studies have focused on the seriousness of moral distress because it directly affects the quality of patient care; it reduces nurses’ job satisfaction, increases employee turnover, and causes moral desensitisation that reduces their moral sensitivity [26,27]. However, few studies have identified the degree of moral distress and verified its influence on job satisfaction with nurses acting as PAs. 

Therefore, the purpose of this study was to identify (1) the levels of professional autonomy, nurse–physician collaboration, moral distress, and job satisfaction; and (2) the impact of these four variables among nurses acting as PAs in South Korea.

## 2. Materials and Methods

### 2.1. Participants

A descriptive survey design [28] was selected to represent the population of nurses acting as PAs from five general hospitals in three major regions in South Korea: Seoul, Gyeonggi, and Gangwon Province, through a convenience sample (N = 148). Although a convenience sampling was employed, the general hospitals selected have diverse departments, including general, critical, and some special units, with at least 300 full-time registered nurses (RNs). The required sample size was calculated considering a significance level of α = 0.05, power set at 80%, and medium effect size = 0.15; the number of relevant variables was determined using the G*power Program (3.1 version, IBM Korea, Seoul, Republic of Korea). Adding a dropout rate and an error range of 20% resulted in a necessary sample size of 148. 

The 148 participants were assessed between 1 August and 30 September 2017, and the questionnaires were distributed to nurses acting as PAs except for nurse practitioners (NPs); the focus was on staff RNs who were directly placed into the PA without specialised education or training or advanced qualification. Moreover, in South Korea, most nurses acting as PAs consist of staff RNs and not NPs [5]. The participants were asked to seal the questionnaires in an envelope for confidentiality, and the researcher collected the questionnaires during visits to the hospitals or by mail. Only 135 questionnaires were returned, for a response rate of 91.2%. After excluding ambiguous and missing responses, 130 copies were used for analysis.

### 2.2. Instruments

#### 2.2.1. General Characteristics

The questions for measuring the general characteristics of the participants were composed based on previous studies [11,12]; they were organised into seven items: gender, age, marital status, religion, educational level, workplace, and years of nursing experience.

#### 2.2.2. Job Satisfaction 

A Korean version of the Minnesota Satisfaction Questionnaire (K-MSQ) was employed, which was validated by Yoon [29] with the Minnesota Satisfaction Questionnaire (MSQ) of Weiss et al. [30]. The author of the Korean version granted permission to use the K-MSQ. It is composed of 19 items rated on a 5-point Likert scale that ranges from 1 (not at all) to 5 (very much so). A higher score indicates greater job satisfaction. Weiss, Dawis and England [30] reported an MSQ reliability of 0.81. In this study, the K-MSQ reliability was generated with Cronbach’s alpha of 0.78.

#### 2.2.3. Nurse–Physician Collaboration 

A Korean version of the Nurse–Physician Collaboration Scale (K-NPCS) was employed, which was validated by Mun [31] from the Nurse–Physician Collaboration Scale (NPCS) developed by Ushiro [32]. Permission to use the K-NPCS was granted by Dr. Ushiro and the author of the Korean version. The K-NPCS contains 27 items divided into three subscales: sharing information about patients, participating in decision-making concerning patient care, and cooperativeness; specifically, the operational definition of cooperativeness was ‘providing comprehensive care to patients from a patient-centred perspective’ [32]. The items were scored using a 5-point Likert scale (1 = always, 2 = usually, 3 = sometimes, 4 = rarely and 5 = never) and the total score ranges from 27 to 135. A lower score indicates a higher level of nurse–physician collaborative behaviour. Ushiro reported a Cronbach’s alpha reliability of 0.85 [32], and Mun found a reliability of 0.95 [31]. In this study, the K-NPCS reliability was generated with Cronbach‘s alpha of 0.85.

#### 2.2.4. Moral Distress

A Korean version of the Moral Distress Scale-Revised (KMDS-R) was employed, which was validated by Chae et al. [33] from the Moral Distress Scale-Revised (MDS-R) developed by Hamric et al. [34] for nurses. Permission to use the KMDS-R was granted by Dr. Hamric and Dr. Chae. The KMDS-R is a 21-item scale consisting of the situations causing moral distress in five subdomains for ’futile care’, ’nursing practice’, ’institutional and contextual factor’, ’physician practice’, and ’limit to claim the ethical issue’. A total of 21 questions, consisting of a 5-point Likert scale, were developed to measure the frequency of moral distress (0: *‘never’*–4: *‘very frequently’*) and the intensity of moral distress (0: *‘none’*–4: *‘great extent’*). The moral distress score was calculated by multiplying the frequency score and intensity score of the distress by ‘0’ if you have never experienced or have no intensity in any item. The number of points of moral distress was calculated as the total score by adding all the scores of each question; a higher score indicates a stronger sense of moral distress. Hamric, Borchers and Epstein [34] reported a Cronbach’s alpha reliability of 0.89, and Chae, Yu, Lee and Park [33] found a reliability of 0.91. In this study, the KMDS-R reliability was generated with a Cronbach’s alpha of 0.85.

#### 2.2.5. Professional Autonomy 

A Korean version of the Schutzenhofer Professional Autonomy Nursing Scale (K-SPANS) was employed, which was validated by Yang [35] with the K-SPANS of Schutzenhofer [36]. The author of the Korean version granted permission to use the scale. Each item describes a situation in which a nurse would have to exercise some degree of autonomy. The scale asks participants to respond to each item even if they never have or may never encounter the situation. The following Likert scale format was used to answer each item: (1) ‘very unlikely of me to act in this manner’, (2) ‘unlikely of me to act in this manner’, (3) ‘likely of me to act in this manner’, and (4) ‘very likely of me to act in this manner’ [36]. Responses were weighted to measure three levels of autonomy (1 = low level of autonomy to 3 = high level of autonomy). The adjusted scores were then combined, resulting in a score range from 60 to 240. Professional autonomy scores range from 60 to 120 for the low level, from 121 to 180 for the moderate level, and from 181 to 240 for the high level [36]. A total weighted score of the SPANS was calculated. Schutzenhofer reported a Cronbach’s alpha reliability of 0.92 [36], and Yang found a reliability of 0.83 [35]. In this study, the K-SPANS reliability was generated with a Cronbach’s alpha of 0.83.

### 2.3. Data Analysis

Using IBM SPSS for Windows v. 21.0 (IBM, New York, NY, USA), Cronbach’s alpha reliability analysis, t-test, one-way analysis of variance (ANOVA), Pearson’s correlation coefficients, and multiple regression analysis were conducted.

### 2.4. Ethical Considerations

The study was conducted in accordance with the Declaration of Helsinki and was approved by the Institutional Review Board of Hallym University before data collection (HIRB NO. 2017-047). Written informed consent about the study purpose, including guaranteed anonymity and confidentiality, was obtained; only the participants who voluntarily agreed to participate were sampled, and any participant could withdraw at any time without repercussions. When participants could not deliver the questionnaire to the researcher directly due to their work shift, they were asked to seal it in an envelope for confidentiality. 

## 3. Results

### 3.1. General Characteristics and Differences in Job Satisfaction According to General Characteristics

Of all the participants, 47.7% were between 20 and 29 years, with a mean age of 31.38 years (SD = 5.58). A total of 108 respondents were female, and over half did not have any religion (60.8%) and had bachelor or graduate degrees (77.7%). The mean years of nursing experience was 7.72 years (SD = 5.98). There were no differences in job satisfaction according to general characteristics (Table 1).

### 3.2. Levels of Job Satisfaction, Nurse–Physician Collaboration, Moral Distress, and Professional Autonomy

The mean for job satisfaction was 2.86 (SD = 0.90). The mean for collaboration was 101.95 (SD = 15.87). In the three subdomains, the highest mean was for the subdomain ’Sharing patient information’ (Mean = 4.01, SD = 0.51), and the lowest was for ’Cooperativeness’ (Mean = 3.63, SD = 0.85; Table 2). The mean for moral distress was 87.50 (SD = 59.65). In the five subdomains, the highest mean was for the subdomain ’Futile care’ (Mean = 6.10, SD = 4.32) and the lowest was for ’Physician practice’ (Mean = 2.65, SD = 3.39). The mean for professional autonomy was 158.66 (SD = 21.19). Nurses with moderate levels of professional autonomy were highest, and nurses in the high-level group were lowest (n = 117, 90.00% and n = 5, 3.85%, respectively). 

### 3.3. Correlations among Job Satisfaction, Nurse–Physician Collaboration, Moral Distress, and Professional Autonomy

Job satisfaction was negatively correlated with moral distress (r = −0.450, *p* < 0.001), but positively correlated with professional autonomy (r = 0.457, *p* < 0.001) and nurse–physician collaboration (r = 0.338, *p* < 0.001). The correlations between all subdomains of moral distress and nurse–physician collaboration and job satisfaction were significant (Table 3). 

### 3.4. Impact of Nurse–Physician Collaboration, Moral Distress, and Professional Autonomy on Job Satisfaction

This section identifies the impact of moral distress, professional autonomy, and collaboration on job satisfaction using a stepwise multiple regression analysis. In the final regression model, the adjusted R square was significant, explaining 38.2% of the variance of job satisfaction (F = 8.303, *p* < 0.001) when ‘cooperativeness’ (β = 0.469, *p* = 0.001) from nurse–physician collaboration, ‘institutional and contextual factor’ from moral distress (β = −0.292, *p* = 0.014), and professional autonomy (β = 0.247, *p* = 0.015) were included (Table 4). The stepwise regression model was evaluated for multicollinearity. The Durbin–Watson statistic was 1.857, close to 2.0, indicating no autocorrelation in the residuals, and the variance inflation factor (VIF) was 1.314–3.267 (smaller than 10) and did not present multicollinearity concerns [37].

## 4. Discussion

Nurse–physician collaboration was the most significant predictor of job satisfaction in our study, consistent with other research findings [38,39]. Notably, cooperativeness was one of the significantly influential factors on job satisfaction among nurses acting as PAs. This finding could be interpreted to suggest that nurses acting as PAs may be satisfied with their job when they are able to deliver personalised care through teamwork, because the operational definition of cooperativeness is ’providing comprehensive care to patients from a patient-cared perspective’ defined by the original instrument’s author, Dr. Ushiro [32]. This was also found in a recent literature review on job satisfaction among PAs in other countries [40]. Edvardsson et al. [41] also found a significantly positive correlation between person-centred care (PCC) and job satisfaction among aged care staff in Australia, and they suggested that supporting staff in providing PCC can enhance job satisfaction. However, in a recent scoping review on PCC and job satisfaction among healthcare providers, the association was significantly positive in cross-sectional studies, while there was no association in longitudinal studies [42]. Even though most studies have focused on PCC in a patient-related context, further studies should examine the association between collaboration based on PCC and healthcare providers’ job satisfaction. 

Moreover, the participants perceived ’cooperativeness’ as lowest among the subdomains of nurse–physician collaboration. This finding is difficult to compare with results from other studies as most studies on this impact on job satisfaction reported a single item [38,39]. This finding could be caused by the ambiguous PA system in South Korea [6,43]; the balance in authority between nurses acting as PAs and their physicians is not equal, because nurses have no choice but to learn and practice from their individual physician, as the medical residents’ apprentices do in didactic education. For this reason, nurses acting as PAs are sometimes likely to be regarded not as nurses but as physician residents in their hospitals. However, those nurses will have to return to being general nurses someday; thus, they often get confused about their professional identity [5,6]. After nurses acting as PAs undertook specialised programs from their institutions, their job satisfaction increased as they could now redefine their role [7]. It is no surprise that there has been a constant demand for improvement and development of such a system and training of PAs even in countries where they legally operate as PAs [40,44]. 

In our findings, it is noteworthy that moral distress resulted from institutional and contextual factors, which can predict decreased job satisfaction among nurses acting as PAs, which is consistent with other studies regardless of nurses’ care settings or cultural backgrounds [45,46]. The consistency can be explained by ’the crescendo effect model’ in Epstein and Hamric’s terms [47], which they explained on the premise of Webster and Baylis’s concept of moral residue [48]. They described ’moral residue’ as what one continues to feel after the morally problematic situation has passed. The unresolved moral distress can be accumulated repeatedly, to never disappear and simply remain and grow. When a nurse experiences moral distress recurrently, the point of moral distress starts at a higher level than before as a function of moral residue. The more the moral distress is repeated, the more it escalates. This model is named ‘the crescendo effect’. There has been evidence for this model in nursing reviews on moral distress over the last decade [21,24,25,49]. 

As shown in this study, moral distress is caused by increased situational constraints and can hardly be solved by individual nurses unless the system itself is changed, and job satisfaction needs to be recognised urgently [11,26]. According to Kim, Oh and Kong’s phenomenological study on ethical conflicts among nurses [26], when nurses were repeatedly confronted with unmanageable moral distress, they felt helpless and incompetent in their hospitals, resulting in them leaving their jobs. In their study, we should note that a few remaining nurses showed moral indifference or desensitisation, impacting nursing care and patients’ outcomes. Hamric and Epstein [50] proposed a moral distress consultation service, which can create an ethical environment for healthcare providers to work through in order to continue to provide safe, high-quality care while maintaining their moral integrity in the process. 

Meanwhile, no significant association was found between moral distress and job satisfaction in a study conducted with 142 critical care nurses in Iran [51]. In this context, the most influential factor of job satisfaction seemed to be the salary. The gap might originate from different healthcare environments because both intrinsic (e.g., moral distress) and extrinsic factors (e.g., salary) have been emphasised by nursing researchers as essential to job satisfaction [11,52]. In contrast, it was hard to compare our finding to Iranian PAs as there has been little research on the moral distress of PAs. Therefore, organisations should investigate and reduce the institutional and contextual factors causing moral distress experienced by nurses acting as PAs. Further research is needed on the relationship between nurses’ moral distress and job satisfaction in various healthcare environments, and similar research for PAs is suggested. 

In our findings, professional autonomy may significantly predict job satisfaction among nurses acting as PAs, consistent with comprehensive literature reviews in a range of contexts, such as with nurses in different care settings [52,53] and PAs worldwide [44,54]. It can be supported by self-determination theory (SDT), which identifies three basic psychological needs—autonomy, competency, and relatedness—as determinants of satisfaction [55]. According to SDT theory in human resource development, autonomous workers greatly value performing work well when it is work that they want to do; they can be highly motivated when pursuing goals and valuing what is personally meaningful in their judgement, rather than extrinsic factors such as the salary [55,56]. Recently, SDT theory has been applied to understand professional autonomy and satisfaction among healthcare professionals [57,58].

Indeed, the outcomes of professional autonomy motivate nurses to apply their knowledge and skills and foster professionalism; thus, in literature reviews [53,59], it has been found to enhance job satisfaction, commitment, retention of nurses, and clinical outcomes for patients. Conversely, Mastekaasa [60] reported no relationship between autonomy and job satisfaction among physicians, nurses, teachers, and social workers in Norway. This contrast might be caused by sociodemographic differences or the use of psychometric instruments with fewer questions in Mastekaasa’s study (autonomy: 3 items and job satisfaction: 1 item). Moreover, as each country may have a different role for nurses, as required by their respective healthcare systems, further research considering this would also be needed. Further studies to investigate the association between variables in various settings or healthcare systems, controlling for confounding variables such as socio-demographics, are suggested.

Meanwhile, the mean for professional autonomy among nurses acting as PAs was remarkably lower than staff nurses’ in similar cultural contexts (e.g., Japan [61] and China [62]) and also in Western contexts [63,64] when comparing the findings on the same instrument. This could be attributed to confusion about professional identity among Korean nurses acting as PAs [5,6]. In nursing practice, the concept of professional autonomy has been central for the last few decades when based on ethical attributes of nursing knowledge; nurses must act rightly following their ethical values and beliefs, being strictly free from external sources, including other professionals, third parties or organisations [27]. Moreover, in modern society, professional autonomy has been emphasised in multidisciplinary healthcare environments; the public expects each healthcare professional to deliver high-quality care and uphold high ethical standards. To date, numerous nurses acting as PAs in hierarchical and bureaucratic atmospheres in various hospital settings experience significant role conflicts, which is likely to erode their professional autonomy [4,5,6]. Further research needs to focus on the establishment of a policy and system for PAs through legislation to improve their professional autonomy. 

### 4.1. Implications

This research makes several contributions. First, our study verified moral distress as a contributing factor to decreased job satisfaction in the nursing context. Theoretically, in line with Dr. Jameton’s moral distress concept [20,21], situational constraints were confirmed as the cause of moral distress. Second, concerning job satisfaction, the predictabilities of the subdomain in nurse–physician collaboration and moral distress were identified to make our inferences. Notably, while collaborating with physicians, patient-centred care was recognised by nurses acting as PAs as most valuable. Lastly, our study notably illuminated the adverse outcomes resulting from a vulnerable work environment where nurses act as PAs, suggesting the need to create a healthy environment that could directly result in better patient outcomes.

### 4.2. Limitations

This research had two limitations. First, a convenience sampling was employed, and therefore, generalizability is weak. Further, most participants were female because Korean male nurses accounted for only 5.1% of RNs in 2019 [3]. A separate research study on work environments and ethical aspects recognised by male nurses or male nurses acting as PAs may be conducted for comparison. Second, our data were obtained using self-report questionnaires. Some participants might underreport their moral distress, due to negative feedback from their organization, although our questionnaires were anonymous and confidential. However, this concern can be lessened when psychometrically sound measures are employed [65]. 

## 5. Conclusions

The present study revealed the significant influential factors of job satisfaction among nurses who work as PAs in hospital settings. To improve job satisfaction among this group, cooperativeness between nurses and physicians is vital, and it should be based on the acknowledgement of and respect for nursing’s central value, patient-centredness. In addition, the monitoring and management of moral distress resulting from situational constraints should be understood, as well as the amelioration of the same initiated by organisations. Enhancing their professional autonomy could motivate nurses to revalue their work, resulting in job satisfaction and, ultimately, renewed positive contributions to patient outcomes.

## Figures and Tables

**Table 1:** General characteristics and differences in job satisfaction according to general characteristics (n = 130).

Characteristics	Categories	n (%) or Mean (SD)		Job Satisfaction			
				Mean	(SD)	t or F	*p*
Gender	Female	108	(83.1)	55.41	(7.79)	0.106	0.916
	Male	22	(16.9)	55.19	(8.86)		
Age(year)	20–29	62	(47.7)	55.32	(8.58)	0.007	0.993
	30–39	56	(43.1)	55.14	(8.58)		
	40–49	12	(9.2)	55.17	(10.21)		
		31.38	(5.58)				
Marital status	Married	42	(32.3)	56.17	(10.10)	−0.850	0.397
	Unmarried	88	(67.7)	54.78	(7.91)		
Religion	With a religion	51	(39.2)	54.31	(9.58)	0.969	0.334
	Without a religion	79	(60.8)	55.82	(8.03)		
Educational level	Associate’s degree	29	(22.3)	55.59	(8.15)	0.404	0.669
	Bachelor’s degree	79	(60.8)	54.73	(8.99)		
	Graduate school	22	(16.9)	56.55	(8.34)		
Workplace	Internal medicine ward	42	(32.3)	56.02	(8.14)	1.215	0.300
	Surgical medicine ward	86	(66.2)	55.05	(8.85)		
	General medicine ward	2	(1.5)	46.50	(10.61)		
Years of nursing experience	<3 years	30	(23.1)	54.40	(8.56)	0.633	0.640
	3~<5 years	22	(16.9)	55.77	(9.80)		
	5~<10 years	42	(32.3)	56.62	(5.75)		
	10~<15 years	21	(16.2)	53.24	(12.04)		
	>15 years	15	(11.5)	55.00	(8.85)		
		7.72	(5.98)				

**Table 2:** Levels of job satisfaction, nurse–physician collaboration, moral distress, and professional autonomy (n = 130).

Variables	n(%) or Mean (SD)	Range	Min–Max
Job satisfaction	2.86(0.90)	1–5	1.00–5.00
Nurse–physician collaboration	101.95(15.87)	27–135	56.00–135.00
Sharing patient information	4.01(0.51)		3.00–5.00
Decision-making process	3.65(0.79)		2.00–5.00
Cooperativeness	3.63(0.85)		1.00–5.00
Moral distress	87.50(59.65)	0–336	0.00–277.00
Futile care	6.10(4.32)		0.00–15.00
Nursing practice	5.70(3.88)		0.00–16.00
Institutional and contextual factor	5.13(3.88)		0.00–16.00
Limit to claim the ethical issue	3.04(2.70)		0.00–12.00
Physician practice	2.65(3.39)		0.00–16.00
Professional autonomy	158.66(21.19)	60–240	89.00–227.00
Moderate level	117(90.00)		
Low level	8(6.15)		
High level	5(3.85)		

**Table 3:** Correlation among job satisfaction, nurse–physician collaboration, moral distress, and professional autonomy (n = 130).

	Job Satisfaction	
	r	*p*
Moral distress	−0.450	<0.001 **
Institutional and contextual factor	−0.503	<0.001 **
Limit to claim the ethical issue	−0.450	<0.001 **
Nursing practice	−0.360	<0.001 **
Physician practice	−0.295	0.001 **
Futile care	−0.229	0.012 *
Nurse–physician collaboration	0.338	<0.001 **
Cooperativeness	0.573	<0.001 **
Sharing patient information	0.493	<0.001 **
Decision-making process	0.398	<0.001 **
Professional autonomy	0.457	<0.001 **

* *p* < 0.01, ** *p* < 0.001.

**Table 4:** Impact of nurse–physician collaboration, moral distress, and professional autonomy on job satisfaction.

Independent Variables	Unstandardised Coefficient		Standardised Coefficients			Collinearity Statistics	
	B	S.E.	β	t	*p*	Tolerance	VIF
(constant)	−2.256	1.067		−2.115	0.037		
Nurse–physician collaboration							
Cooperativeness	1.339	0.374	0.469	3.583	0.001	0.306	3.267
Moral distress							
Institutional and contextual factor	−0.012	0.005	−0.292	−2.494	0.014	0.381	2.622
Professional autonomy	0.013	0.005	0.247	2.465	0.015	0.521	1.919

F = 8.303, *p* < 0.001, *R^2^* = 0.435, Adjusted *R^2^* = 0.382, *d*(*d_u_*) = 1.857.

## Data Availability

Data can be made available upon request from the corresponding author of the study.
